# Different expression levels of glycans on leukemic cells—a novel screening method with molecularly imprinted polymers (MIP) targeting sialic acid

**DOI:** 10.1007/s13277-016-5280-y

**Published:** 2016-07-31

**Authors:** Zahra El-Schich, Mohammad Abdullah, Sudhirkumar Shinde, Nishtman Dizeyi, Anders Rosén, Börje Sellergren, Anette Gjörloff Wingren

**Affiliations:** 1 Department of Biomedical Sciences, Faculty of Health and Society, Malmö University, Malmö, Sweden; 2 Department of Translational Medicine, Lund University, Malmö, Sweden; 3 Department of Clinical and Experimental Medicine, Division of Cell Biology, Linköping University, Linköping, Sweden

**Keywords:** Chronic lymphocytic leukemia, Lectin, Molecular imprinting polymers, Sialic acid

## Abstract

Sialic acid (SA) is normally expressed on the cell membranes and is located at the terminal position of the sugar chains. SA plays an important role for regulation of the innate immunity, function as markers of the cells and can be recognized by a variety of receptors. Interestingly, the level of SA expression is increased on metastatic cancer cells. The availability of specific antibodies against SA is limited and, therefore, biomarker tools for detection of SA are lacking. We have recently presented a novel method for specific fluorescence labeling of SA molecular imprinted polymers (MIP). Here, we have performed an extended screening of SA expression by using SA-MIP and included four different chronic lymphocytic leukemia (CLL) cell lines, conveniently analyzed by flow cytometry and fluorescence microscopy. SA expression was detected in four cell lines at different levels, and the SA expression were verified with lectin-FITC. These results show that SA-MIP can be used as a plastic antibody for detection of SA using both flow cytometry and fluorescence microscopy. We suggest that SA-MIP can be used for screening of different tumor cells of various stages, including CLL cells.

## Introduction

Glycans, or polysaccharides, are present on the outer surface of most eukaryotic cells and are involved in various cell processes, including protecting the cells from intruders and in cellular connection. Attachment of *O-* and *N-*linked glycans to proteins for the generation of glycoproteins is very common structural motifs [1]. Sialic acid (SA) plays an important role for regulation of the innate immunity and may have an inhibitory effect on complement activation [2]. SA’s attached at the end of glycan chains provides a negative charge on the cell surface, which is required for trafficking of cells. SA functions as a cellular marker and can be recognized by a variety of receptors [3]. In the late 80s, an association between SA and cancer was discovered, especially on metastatic cancer [4, 5]. Fuster reported that overexpression of SA in metastatic cancer controls tumor cell growth and differentiation by interfering with neural cell adhesion molecule signaling at cell-cell contacts [1, 6]. Overexpression of tumor-associated SA in metastatic bladder tumors has been shown, and it was speculated whether there is a benefit of the combination of anti-SA immunotherapy and anti-proliferative drugs [7].

Analyzing and determining these glycosylation motifs is therefore an important diagnostic goal but the task is a challenge due to the limited availability of appropriate lectins and glycan-specific antibodies [8, 9]. This calls for the development of alternative glycan-specific probes and cell imaging technologies. Recent reports have shown that synthetic probes based on host guest chemistry [10] or molecular imprinting technology [11] can be used for glycan selective staining of cells and tissues. Using the former approach, Xu and colleagues developed fluorescent sensors based on two boronic acids linked to a functionalized peptide for detection of cell-surface cancer-associated glycans [10]. On the other hand, imaging probes based on phenylboronic acid-modified quantum dots have also been used, allowing specific labeling of SA on living cells [12]. However, this approach has the weakness of targeting diols in a non-specific manner.

Molecular imprinting, as a design concept, is in this context more attractive. Artificial receptors are produced by allowing a network polymer to assemble in the presence of a template. After removing the template, the resulting molecularly imprinted polymer (MIP) can be used as an artificial antibody [13]. A key advantage of this technique is that it can be used for recognizing less immunogenic targets such as glycans [14]. Moreover, compared with antibodies, MIPs can be produced at low cost and feature superior robustness and long-term stability [15].

Recently, different monosaccharide imprinting procedures were used to produce fluorescently labeled nanoparticle probes displaying an unprecedented affinity for the targeted terminal monosaccharides in cell staining experiments [16]. Based on a ternary complex imprinting approach, we developed imprinted core-shell nanoparticles showing an exceptional affinity for cell surface SA-glycans. The probe comprised a nitrobenzoxadiazole (NBD) fluorescent reporter group showing enhanced emission intensity in presence of the SA-guest. In flow cytometry and fluorescence microscopy investigations, the SA-MIP selectively targeted two different prostate cancer cell lines DU145 and PC3, as well as the T leukemia cell line Jurkat. The SA expression was verified with lectin-FITC [11].

In this study, we have performed an extended screening of SA expression by using SA-MIP using four different chronic lymphocytic leukemia (CLL) cell lines, conveniently analyzed by flow cytometry and fluorescence microscopy. Indeed, SA expression was detected in all four cell lines and at different levels.

## Material and methods

### Cell culture

Four CLL cell lines were used, HG3, CI, Wa-osel, and AIII [17]. The cell lines were cultured in RPMI-1640 medium (Invitrogen, San Diego, CA, USA) supplemented with 10 % fetal bovine serum (FBS, Invitrogen) and 50 μg/mL gentamycin (Invitrogen) and incubated in 37 °C with 5 % CO_2_ in 100 % humidity.

### Preparation of polymers

The polymers (SA-MIP) were prepared as described previously [11]. In brief, the dried SA-MIP were resuspended in water/water and analyzed using (3 % methanol, Sigma-Aldrich Co., St. Louis, MI) and sonicated for 8 min with a VWR sonicator. The stock solution of 0.8 mg/ml was further sonicated and diluted prior to use.

### Flow cytometry analysis of SA-MIP and lectin-FITC

1 × 10^6^ cells/sample were stained with either SA-MIP, lectin-FITC (Sigma-Aldrich) or left unstained as a control. The cells of each CLL cell line were washed twice with 2 ml phosphate-buffered saline (PBS, Invitrogen), and fixed with 1 ml of 4 % formaldehyde (Sigma-Aldrich) and incubated for 20 min at room temperature (RT). After fixation, the cells were washed three times with 2 ml PBS and, thereafter, two times with 2 ml 3 % methanol. Five hundred microliters of a sonicated polymer suspension of concentrations of 400, 200, 80, and 40 μg/ml, respectively, was added to the cells, and 500 μL of 3 % methanol was used as a negative control. The cells were incubated with SA-MIP for 90 min in 37 °C and were thereafter washed three times with 2 ml 3 % methanol/water and analyzed using flow cytometry (BD Biosciences, Accuri C6 Flow Cytometry, NJ).

For lectin staining, cells were incubated with 500 μl of lectin-FITC of concentrations 100, 50, 25, and 10 ng/ml or left unstained as a negative control with 500 μl PBS. The cells were incubated in the dark for 20 min, on ice, and were thereafter washed three times with 2 ml PBS and analyzed by flow cytometry.

### Ligand binding assay of SA based on flow cytometry

A ligand binding assay based on the flow cytometry analysis, i.e., quantification of cellular fluorescence of the CLL cell lines was performed. The saturation capacity of the binding sites in the SA-MIP was 10 μmol g^−1^ [11]. The binding curve was fitted by non-linear regression to a Langmuir mono-site model using the Prism 6 curve fitting software (Graph pad Inc.).

### Fluorescence microscopy of SA-MIP and lectin-FITC

Fifty thousand cells/sample of HG3 cells were adhered to poly-lysine treated slides (Thermo Scientific, MA) for 2 h, at 37 °C with 5 % CO_2_ in 100 % humidity. After the incubation, the cells were washed three times with PBS and fixed with 100 μl 4 % formaldehyde for 10 min. Thereafter, the cells were washed three times with PBS and two times with 3 % methanol, and the cells were incubated with 100 μl sonicated SA-MIP at concentration of 100 μg/ml or left unstained with 100 μl 3 % methanol at 37 °C for 60 min. After the incubation, the cells were washed four times with 3 % methanol and, thereafter, twice with PBS, incubated with 300 nM DAPI in PBS (Thermo Fisher Scientific, Carlsbad, CA), and incubated for 4 min at RT. After three more washes with PBS, the cells were mounted with one drop of mounting medium Prolong® Gold antifade reagent (Molecular probes).

For lectin staining, HG3 cells were adhered to poly-lysine-treated slides and, thereafter, fixed with 1 ml 4 % formaldehyde, for 10 min at RT and, thereafter, washed three times with PBS. The cells were incubated with 100 μl of lectin-FITC of concentration 100 ng/ml or left unstained as a negative control with 100 μl PBS. The cells were incubated with lectin-FITC at RT for 60 min. Next, the cells were washed three times with PBS and then incubated with 300 nM DAPI in PBS and incubated for 4 min at RT. After three washes with PBS, the cells were mounted with one drop of mounting medium Prolong® Gold antifade reagent. The stained cells were then analyzed using fluorescence microscopy (EVO® LS 10, Carl Zeiss, Jena, Germany).

## Results

### Expression of SA in the four CLL cell lines HG3, CI, Wa-osel, and AIII as detected by flow cytometry

**Fig. 1:**
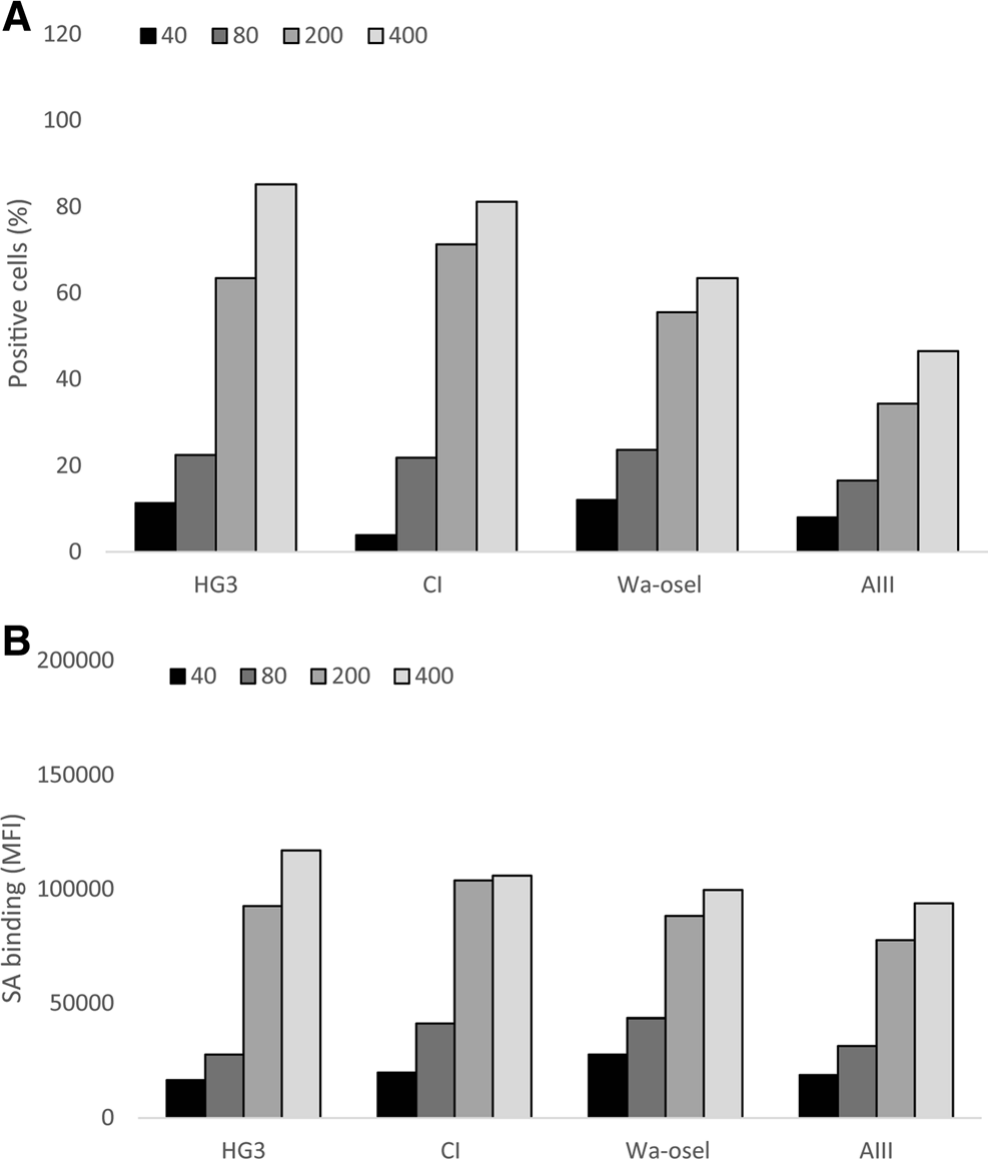
SA expression on the four CLL cell lines. Results of HG3, CI, Wa-osel, and AIII cells stained with different concentrations of SA-MIP. Flow cytometry results present **a** the positive cells for SA and **b** the MFI of the SA binding. One representative experiment out of two performed is shown

**Fig. 2:**
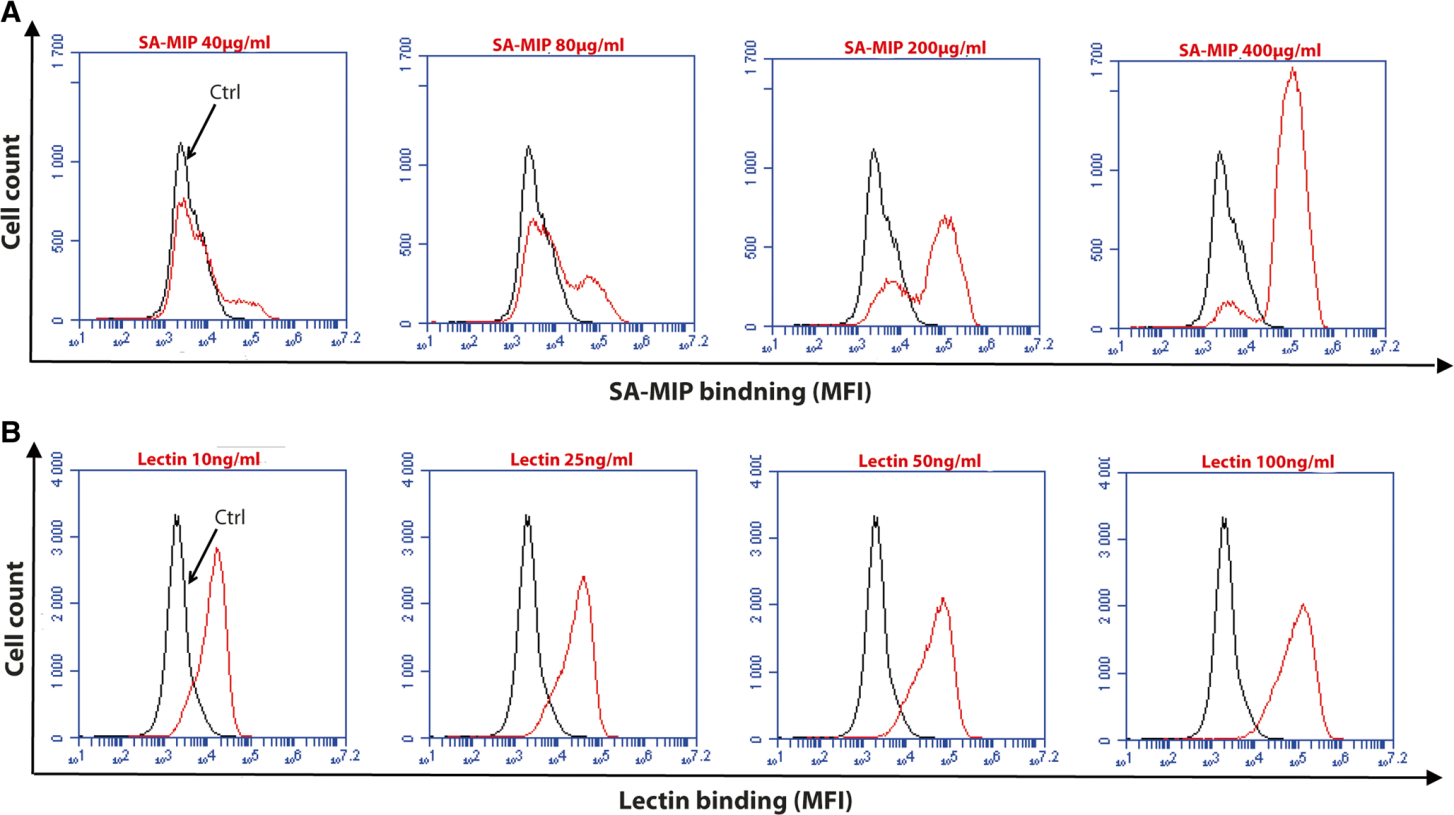
SA and lectin binding on HG3 cells. Flow cytometry analysis of HG3 cells with different concentrations of SA-MIP (**a**) and lectin-FITC (**b**). *Black traces* show the unstained samples, while the *red traces* show SA-MIP (**a**) and lectin-FITC (**B**). The results are presented as MFI. One representative experiment out of two performed is shown

**Fig. 3:**
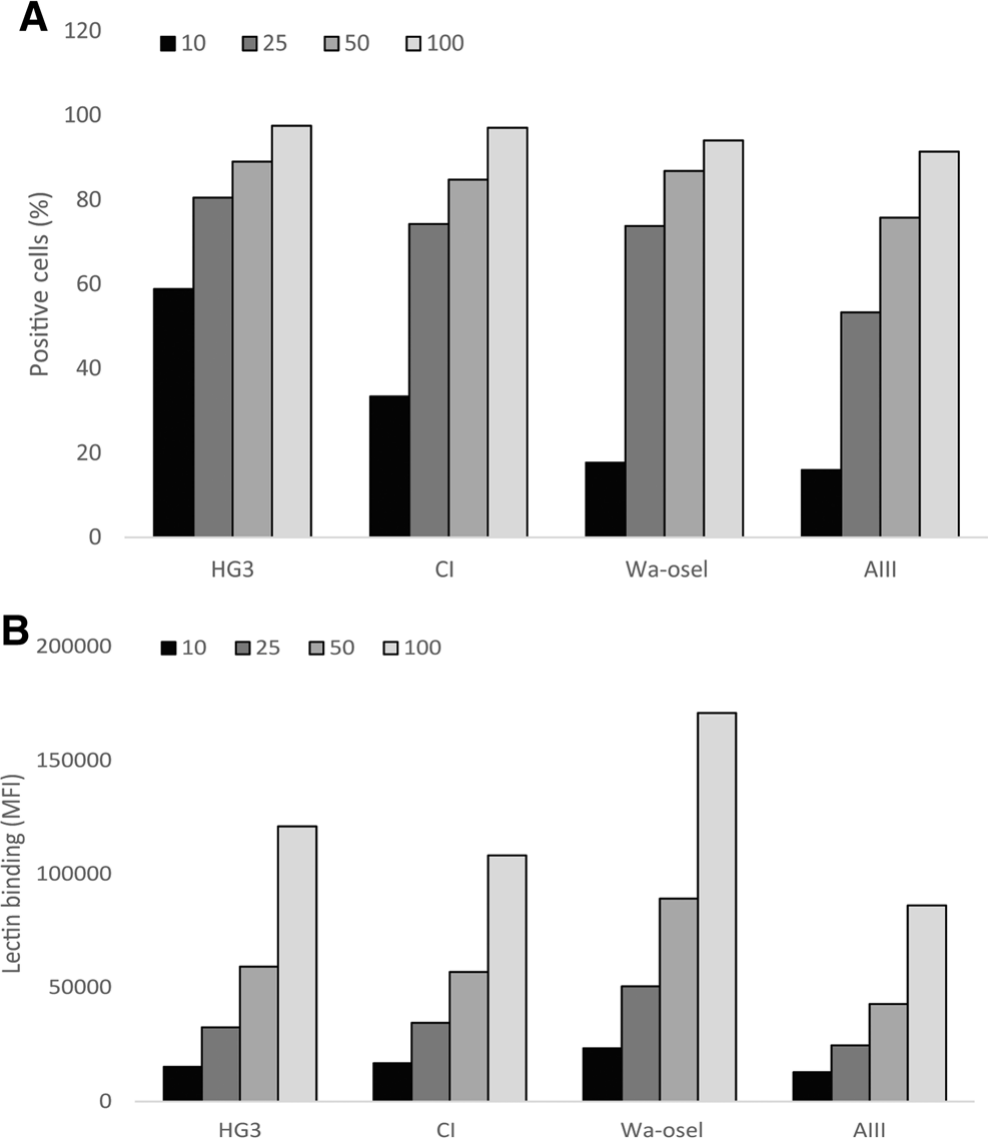
Lectin binding on the four CLL cell lines. Results of HG3, CI, Wa-osel, and AIII cells stained with different concentrations of lectin-FITC. Flow cytometry results present **a** the positive cells for lectin binding and **b** the MFI of the lectin binding. One representative experiment out of two performed is shown

The surface expression of SA in the four CLL cell lines was investigated by flow cytometry. The fluorescence intensity of the cell lines was different after staining with SA-MIP (200 and 400 μg/ml) (Fig. 1a). HG3 and CI showed a slightly higher percentage of positive cells compared to the cell lines Wa-osel and AIII. When comparing the mean fluorescence intensity (MFI) among the four CLL cell lines, there were however no major differences (Fig. 1b). Figure 2 displays the histogram for the HG3 cell line with different concentrations of SA-MIP (Fig. 2a) and lectin-FITC (Fig. 2b). An increase in binding for SA-MIP to HG3 could be seen, but the lectin-FITC binding was high even at lower concentrations of the lectin. However, the lectin binding with the 10 ng/ml concentration varied between the CLL cell lines (Fig. 3), being higher for HG3 and CI compared to the cell lines Wa-osel and AIII (Fig. 3a).

### HG3 and CI showed highest specific binding in a ligand binding assay

**Fig. 4:**
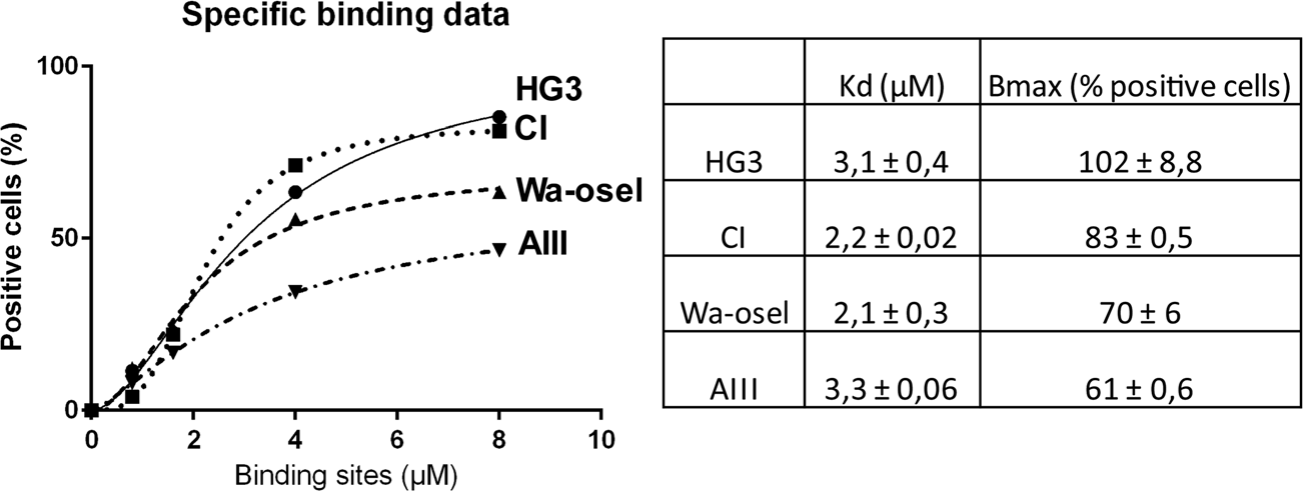
Quantification of cellular fluorescence of the four CLL cell lines. Specific ligand binding assay based on flow cytometry for the four CLL cell lines stained with different concentrations of SA-MIP. For each cell line, the Kd (μM) and B_max_ (% positive cells) are shown

In a saturation ligand binding assay based on the flow cytometry analysis, quantification of cellular fluorescence of the CLL cell lines was possible by using one site specific binding with Hill slope. The specific binding of SA was higher on HG3 and CI compared to Wa-osel and AIII, (Fig. 4).

### SA expression in the HG3 cell line as detected by fluorescence microscopy

**Fig. 5:**
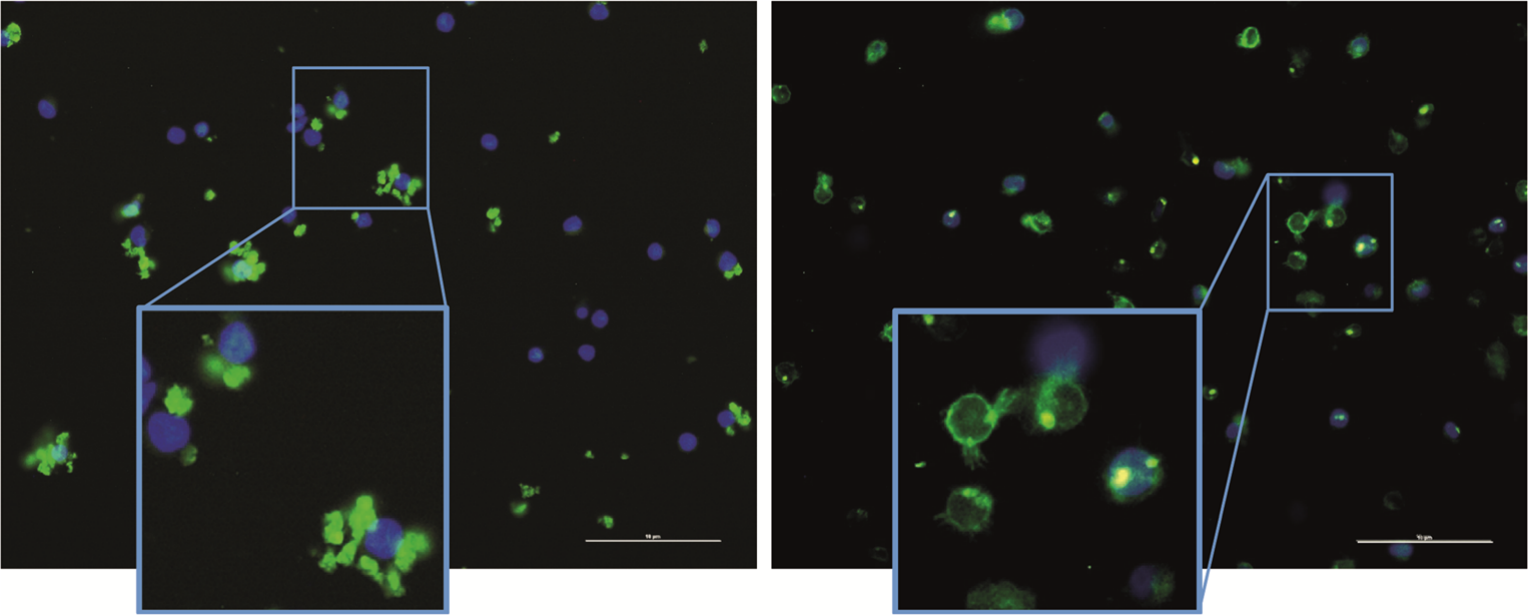
Fluorescence microscopy images of HG3 cells stained with either SA-MIP or lectin-FITC. HG3 cells were stained with either SA-MIP (100 μg/ml, left image) or lectin-FITC (100 ng/ml, *right image*). For nuclear visualization, all samples were stained with DAPI. Scale bar =10 μm

In order to visualize the glycans on the surface of the CLL cell line HG3, the cells were stained with either SA-MIP (Fig. 5a), lectin-FITC (Fig. 5b) or left unstained. All samples were stained with DAPI for nuclear visualization and analyzed with fluorescence microscopy. Overall, the SA-MIP led to a membrane staining of the cells in a qualitatively similar way as lectin-FITC. Staining with lectin-FITC led to a ring-shaped fluorescence pattern all over the cell membrane.

## Discussion

We have previously reported successful cell imaging experiments, where SA-MIP particles selectively stained different cell lines in correlation with the SA expression levels [11]. This was further verified by enzymatic cleavage of SA and by staining using a FITC-labeled lectin. SA plays an important role in a variety of biological processes in cells. In this study, we were primarily interested to verify any correlations with the aggressiveness of the CLL cell lines and SA expression levels [17].

SA expression is enhanced in metastatic cancer cells [18, 19]. CLL cells are CD5+/CD19+ B cells originating from antigen-experienced B1-like B-cells [20, 21]. Indeed, both normal and malignant leukocytes utilize adhesive interactions to migrate throughout the body [22]. Selectins and the sialic-acid-binding immunoglobulin-like lectins (Siglecs) are the intrinsic receptors within vertebrate cells that recognize the SA. Selectins have an important role in the spread of epithelial cancers through the bloodstream. Studies in selectin-deficient mice further confirmed a pivotal role of these glycan-binding proteins in various models of cancer metastasis [23–25]. Siglec-2 (also known as CD22) is a member of the Siglec family, expressed mostly on B cells, recognizes SA as a ligand [26]. The four CLL cell lines used in this study, HG3, CI, Wa-osel, and AIII, all expressed SA at different levels. For each cell line, we show a dose-dependent increase in the number of SA-positive cells as analyzed by flow cytometry. Interestingly, two of the CLL cell lines, HG3 and CI, expressed higher levels of SA compared to the other CLL cell lines, Wa-osel and AIII. This is in line with the fact that the aggressiveness of the cancer cells correlates with increasing SA levels [18, 19]. HG3 and CI have an unmutated immunoglobulin heavy chain variable (*IGHV*) gene, which is associated with high cell proliferation and a more aggressive form of CLL compared with Wa-osel and AIII cells, which have a mutated *IGHV* gene, a signature of less aggressive indolent CLL cells [17].

Analyzing SA on leukocytes can be technically complex, since SA has been shown to be masked by endogenous sialylated ligands [27]. Sialidase treatment or cellular activation is necessary to unmask these sites, possibly by endogenous sialidase efficiency. However, in this study, we could not detect any differences in SA expression after anti-IgM ligation for up to 72 h of the CLL cell lines (data not shown).

Many studies describe changes in glycosylation pattern following neoplastic transformation. Defining the glycan expression of an individual epitope within tissue sections using traditional approaches can be challenging [28, 29]. Improved diagnostics and treatment of cancer is one of the most challenging tasks for researchers today. The transformation from a normal cell into a tumor cell is a multistage process, typically a progression from a pre-cancerous lesion to malignant tumors. Despite the progress in developing new therapeutic modalities, cancer remains one of the leading diseases causing human mortality [30]. Detection of SA has been limited due to the lack of specific antibodies [9]. Here, we have used a highly specific SA-MIP for detection of SA on CLL cell lines. We suggest that SA-MIPs can be used for screening of different circulating tumor cells of various stages, including CLL cells. Further analysis of SA expression should include primary CLL cells from patient samples.

## Conclusions

We have demonstrated SA expression on CLL cell lines with different levels of malignancy by using SA-MIPs. In conclusion, SA-MIPs can be used as plastic antibodies for detection of SA using both flow cytometry and fluorescence microscopy. SA-MIPs have high specificity and affinity for SA in different cell lines. In this context, we could detect differences of SA expression in CLL cell lines.
